# Overexpression of a Brix Domain-Containing Ribosome Biogenesis Factor ARPF2 and its Interactor ARRS1 Causes Morphological Changes and Lifespan Extension in *Arabidopsis thaliana*

**DOI:** 10.3389/fpls.2018.01177

**Published:** 2018-08-27

**Authors:** Shugo Maekawa, Yoshiaki Ueda, Shuichi Yanagisawa

**Affiliations:** Biotechnology Research Center, The University of Tokyo, Tokyo, Japan

**Keywords:** *Arabidopsis thaliana*, Brix domain, lifespan, ribosome biogenesis, yeast Rpf2 homolog

## Abstract

The Brix domain is a conserved domain in several proteins involved in ribosome biogenesis in yeast and animals. In the *Arabidopsis* genome, six Brix domain-containing proteins are encoded; however, their molecular functions have not been fully characterized, as yet. Here we report the functional analysis of a Brix domain-containing protein, ARPF2, which is homologous to yeast Rpf2 that plays an essential role in ribosome biogenesis as a component of the 5S ribonucleoprotein particle. By phenotypic characterization of *arpf2* mutants, histochemical GUS staining, and analysis using green fluorescence protein, we show that *ARPF2* is an essential and ubiquitously expressed gene encoding a nucleolar protein. Co-immunoprecipitation and split-GFP-based bimolecular fluorescence complementation assays revealed that ARPF2 interacts with a protein named ARRS1, which is homologous to yeast Rrs1 that forms a complex with Rpf2 in yeast. Furthermore, the result of RNA immunoprecipitation assay indicated that ARPF2 interacts with 5S ribosomal RNA (rRNA) or the precursor of 5S rRNA, as well as with the internal transcribed spacer 2 in the precursors of 25S rRNA. Most intriguingly, we found that the overexpression of *ARPF2* and *ARRS1* leads to characteristic phenotypes, including short stem, abnormal leaf morphology, and long lifespan, in *Arabidopsis*. These results suggest that the function of Brix domain-containing ARPF2 protein in ribosome biogenesis is intimately associated with the growth and development in plants.

## Introduction

Ribosome biogenesis is a complex process that requires numerous factors, including ribosomal proteins (RPs), ribosomal RNAs (rRNAs), and ribosome biogenesis factors (RBFs) (Henras et al., 2015; Weis et al., 2015a). In eukaryotes, ribosome biogenesis begins with the synthesis of 45S pre-rRNA, which functions as a scaffold for the assembly of RPs, RBFs, and small nucleolar RNAs to form the 90S pre-ribosomal particle in the nucleolus (Henras et al., 2015; Weis et al., 2015a). During the maturation steps, the 45S pre-rRNA in the 90S pre-ribosomal particle is processed into 18S, 5.8S, and 25S rRNA by the elimination of 5′- and 3′-external spacer regions [5′-external transcribed spacer (ETS) and 3′-ETS] and two internal spacer regions [internal transcribed spacer (ITS) 1 and ITS2], and the 90S pre-ribosomal particle is divided into the 40S pre-ribosome subunit, containing 18S rRNA, and the 60S pre-ribosome subunit, containing 5.8S and 25S rRNAs (Henras et al., 2015; Weis et al., 2015a). In the next step, the 5S ribonucleoprotein particle (RNP), comprising two RPs (Rpl5 and Rpl11 in *Saccharomyces cerevisiae*) and 5S rRNA, which is transcribed in the nucleoplasm, assembles into a 60S pre-ribosomal particle (Madru et al., 2015). Finally, the 40S and 60S pre-ribosomal subunits are exported into the cytoplasm and are re-assembled to form the functional mature ribosomes (Henras et al., 2015; Weis et al., 2015a).

The Brix domain (biogenesis of ribosomes in Xenopus domain) is a region of 150–180 amino acid residues in length, which is conserved in several proteins involved in ribosome biogenesis (Eisenhaber et al., 2001). For example, in *S. cerevisiae*, Brx1, Imp4, Ssf1/Ssf2, Rpf1, and Rpf2 contain this domain (Eisenhaber et al., 2001; Wehner and Baserga, 2002). Brx1 was the first Brix domain-containing protein that was identified; it was characterized as an rRNA-interacting protein involved in pre-rRNA processing in *Xenopus laevis* and *S. cerevisiae* (Kaser et al., 2001). Other Brix domain-containing proteins in *S. cerevisiae*, namely Imp4, Ssf1/2, Rpf1, and Rpf2, also bind to pre-rRNAs, and the depletion of Imp4, Ssf1, Rpf1, or Rpf2 results in defects in the processing of pre-rRNA (Lee and Baserga, 1999; Wehner and Baserga, 2002). Crystal analysis of the structures of Rpf2 and its homolog from *Aspergillus nidulans* revealed that Rpf2 tightly binds to a protein called Rrs1 through the N-terminal half of the Brix domain. Moreover, it was determined that the Rpf2–Rrs1 complex binds to 5S rRNA through the C-terminal half of the Brix domain of Rpf2 to form the pre-60S ribosomal subunit together with the 5S RNP (Asano et al., 2015; Kharde et al., 2015; Madru et al., 2015). Although Rpf2 alone can bind to 5S rRNA, Rpf2 was shown to more rigidly bind to 5S rRNA through forming the complex with Rrs1 (Madru et al., 2015). Furthermore, another Brix domain-containing protein, Imp4, was found to bind to U3 small nucleolar RNA and a U3 small nucleolar RNA-associated protein, Mpp10, through its Brix domain and it played a role in the nucleolar processing of pre-18S ribosomal RNA (Lee and Baserga, 1999; Granneman et al., 2003). Therefore, the Brix domain is supposed as a structural hub for interactions with both protein and RNA that are mediated by its N-terminal and C-terminal halves, respectively.

Brix domain-containing proteins likely play key roles in ribosome biogenesis in plants as well as in yeast and animals; however, plant Brix domain-containing proteins have not been well characterized as yet, although a role of *Arabidopsis* duplicated orthologs of yeast Brx1, BRX1-1 and BRX1-2, in pre-rRNA processing (Weis et al., 2015b) and lethality of the function-deficient mutants of the *Arabidopsis* homolog of yeast Ssf1/Ssf2, SNAIL1 (Hao et al., 2017), have been shown. In the current study, we characterized the *Arabidopsis* homolog of Rpf2, which binds to 5S RNP and is involved in ribosome biogenesis in *S. cerevisiae* (Asano et al., 2015; Kharde et al., 2015; Madru et al., 2015). To avoid confusion with RNA PROCESSING FACTOR2 (RPF2) that is required for 5′-end processing of mRNAs in the mitochondria of *Arabidopsis* (Jonietz et al., 2010), the *Arabidopsis* homolog of yeast Rpf2 is referred to as ARPF2 (*Arabidopsis thalliana* homolog of yeast Rpf2: At3g23620), hereafter. Our results indicate that *ARPF2* is an essential gene that is ubiquitously expressed and encodes a nucleolar-localized protein. Moreover, it was also found that ARPF2 interacts with the *Arabidopsis* homolog of yeast Rrs1 (referred to as ARRS1, hereafter) and pre-rRNA in plant cells. These results suggest that ARPF2 and ARRS1 play roles similar to those of yeast Rpf2 and Rrs1 in the ribosome biogenesis. Most significantly, we found that the overexpression of *ARPF2* and *ARRS1* in *Arabidopsis* resulted in short height, abnormal leaf morphology, and an extended period of reproductive growth.

## Materials and Methods

### Phylogenetic Analysis

A phylogenetic tree of 23 Brix domain proteins from *Homo sapiens*, *A. thaliana*, *Oryza sativa*, and *S. cerevisiae* was created using MEGA7 (Kumar et al., 2016). The evolutionary history was inferred using the Neighbor-Joining method (Saitou and Nei, 1987). The evolutionary distances were computed using the Poisson correction method (Zuckerkandl and Pauling, 1965).

### Plant Materials and Growth Conditions

*Arabidopsis thaliana* ecotype Col-0 was used as the wild type, and all T-DNA insertion lines and mutants used in this study were in the Col-0 background. The seeds of two T-DNA insertion lines, SALK_107828 for *arpf2-1* and SAIL_314_A03 for *arpf2-2*, were provided by the Arabidopsis Biological Resource Center (Alonso et al., 2003) and the Syngenta Arabidopsis Insertion Library (Sessions et al., 2002), respectively. The seeds were sterilized and sown on agar plates containing half-strength Murashige and Skoog medium supplemented with 1% sucrose. After a 2-day cold treatment, the plates were placed at 23°C under diurnal light condition (16 h light/8 h dark, approximately 70 μmol m^-2^ s^-1^). After 2 weeks of growth, the seedlings were transferred onto peat containing nutrients (Sakatanotane Co., Yokohama, Japan) and further grown at 23°C under continuous light (∼70 μmol m^-2^ s^-1^).

### Plasmid Construction

To generate plasmids for transient expression assay and for generation of transgenic plants, we obtained ARPF2, ARRS1, RPL4A and PRH75 cDNAs by RT-PCR using RNA from Col-0 seedlings. The sequences of primers used for RT-PCR are listed in **Supplementary Table S1**. The resulting PCR products were cloned into the pENTR/D-TOPO vector (Thermo Fisher Scientific Inc.). To construct the binary plasmids for expression of ARPF2 and ARRS1 fused to the N-terminus of GFP (ARPF2-GFP and ARRS1-GFP), ARPF2 and ARRS1 cDNAs were introduced into a Gateway binary vector, pGWB5, whereas RPL4A and PRH75 cDNAs were introduced into another Gateway binary vector, pGWB6, for the expression of RPL4A and PRH75 fused to the C-terminus of GFP (GFP-RPL4A and GFP-PRH75). The ARPF2 and ARRS1 cDNAs were also introduced into pGWB17 to produce expression vectors for ARPF2-MYC and ARRS1-MYC proteins. Additionally, ARPF2 cDNA was introduced into pB4GWnG to produce an expression vector for ARPF2-nGFP in which ARPF2 was fused to the N-terminal half of GFP (nGFP), whereas ARRS1 cDNA was introduced into pB4GWcG to produce an expression vector for ARRS1-cGFP in which ARRS1 was fused to the C-terminal half of GFP (cGFP). Furthermore, RPL4A cDNA was introduced into pB4nGGW and pB4cGGW to produce expression vectors for nGFP-RPL4A and cGFP-RPL4A in which RPL4A was fused to nGFP and cGFP, respectively. Plasmids, pGWB5/6/17, pB4GWnG, and pB4GWcG, were described earlier by Tanaka et al. (2012). To construct the pARPF2:GUS plasmid in which the *ARPF2* promoter was located upstream of the *GUS* reporter gene, an approximately 1.5 kb DNA fragment containing the promoter and 5′-UTR regions of *ARPF2* was amplified by PCR using genomic DNA of the wild type Col-0 plant and a set of PCR primers (**Supplementary Table S1**). The resulting product was initially cloned into the pENTR/D-TOPO vector, and subsequently transferred into pMDC164 (Curtis and Grossniklaus, 2003). All the PCR products and inserts were verified by DNA sequencing.

### Generation of Transgenic *Arabidopsis* Plants

The wild type plants were transformed with the binary vectors for expression of ARPF2-GFP, ARPF2-MYC, ARRS1-MYC, APUM24-GFP (Maekawa et al., 2018), and GFP-PRH75, and pARPF2:GUS plasmid using the floral dip method (Clough and Bent, 1998) with *Agrobacterium tumefaciens* strain GV3101 (pMP90). The transgenic lines with T-DNA insertion(s) at a single locus were selected, and T3 progenies homozygous for the introduced gene were used for all the experiments. Two independent transgenic lines, *ARPF2-GFP*ox#3 and *ARPF2-MYC*ox#5, were subjected to phenotypic analysis of the plants overexpressing *ARPF2*.

### Histochemical GUS Staining

GUS staining was performed in accordance with the standard protocol (Jefferson et al., 1987), with minor modifications. Various plant tissues were incubated in 80% acetone for 20 min at 4°C, and then washed with 50 mM sodium phosphate buffer (pH 7.0). Thereafter, the samples were submerged into the GUS staining buffer that contained 50 mM sodium phosphate buffer (pH 7.0), 100 mM of 5-bromo-4-chloro-3-indolyl β-D-glucuronide, 2 mM potassium ferricyanide, 2 mM potassium ferrocyanide, 10 mM EDTA (pH 8.0), and 0.1% Triton X-100. The staining reaction was carried out at 37°C in the dark for 4 h, and the samples were then washed with 70% ethanol to remove chlorophyll.

### Subcellular Localization Analysis With Green Fluorescence Protein (GFP) and Split-GFP-Based Bimolecular Fluorescence Complementation (BiFC) Assays

The subcellular localization and split-GFP-based BiFC assays were performed by co-infiltrating *Nicotiana benthamiana* leaves (Kapila et al., 1997; D’Aoust et al., 2009) with derivatives of pGWB5, pB4GWnG, and pB4GWcG and the expression vector for FIB1-mCherry, as described previously (Maekawa et al., 2014). Two or three days after the infection, the GFP fluorescence was observed under a fluorescence microscope (BX51, Olympus Corp.) equipped with a cooled color digital camera (DP80, Olympus Corp.).

### Co-immunoprecipitation Analysis

Co-immunoprecipitation analysis was performed by co-infiltrating *N. benthamiana* leaves (Kapila et al., 1997; D’Aoust et al., 2009) with the expression vectors for ARPF2-GFP and ARRS1-MYC. Three days after the infection, the infected leaves (∼100 mg) were frozen in liquid nitrogen and homogenized with zirconia balls in 400 μl of extraction buffer that contained 25 mM Tris-HCl (pH 7.5), 150 mM NaCl, 0.1% NP40, 10% glycerol, and protease inhibitor cocktail from Sigma-Aldrich Co. LLC (P9599 and 11697498001). The cell lysates were incubated with anti-GFP antibody (MBL Co., Ltd.) and protein A agarose beads (Sigma–Aldrich Co. LLC), and then the proteins interacting with the antibody were eluted with the SDS sample buffer (BioRad, Inc.) and resolved by SDS-PAGE. Following transfer to polyvinylidene difluoride membranes, the proteins were detected with anti-GFP (MBL Co., Ltd., 1:1,000) and anti-MYC (Merck Millipore, 1:1,000) antibodies. In a control experiment, ARRS1-MYC protein was transiently expressed alone or with ARPF2-GFP or GFP-RPL4A in *N. benthamiana* leaves. Immunoprecipitates obtained with anti-GFP antibody in the experiment were analyzed with anti-MYC and anti-GFP antibodies.

### RNA Immunoprecipitation (RIP) Assay

The RIP assay was performed as described previously (Maekawa et al., 2018), except for the fact that immunoprecipitation was performed with an anti-GFP antibody (MBL Co., Ltd.). Quantitative PCR (qPCR) was performed using rRNA-related PCR primers (**Supplementary Table S1**), as described in “Expression Analysis”. The enrichment fold was calculated as the ratio of the RNA level in the eluate to that in the input sample.

### Expression Analysis

Total RNA was prepared from seedlings using an ISOSPIN Plant RNA kit (Nippon Gene Co., Ltd., Japan) with a TURBO DNase kit (Thermo Fisher Scientific Inc.), and reverse transcription was performed with Prime Script RT Master Mix (Takara Bio Inc.). The PCR was performed with a StepOne Plus Real Time PCR System (Thermo Fisher Scientific Inc.) using a KAPA SYBR Fast Quantitative PCR kit (KAPA Biosystems, Inc.). The primers used are listed in **Supplementary Table S1**. The relative gene expression levels were calculated using the ΔΔCT method (Livak and Schmittgen, 2001) and normalized relative to the expression levels of *UBQ10*.

### Quantification of Chlorophyll and Cell Death

Chlorophyll quantification was performed with a batch of three to five rosette leaves, as described by Porra et al. (1989). Cell death was quantified by electrolyte leakage. Leaf disks (4 mm diameter) of rosette leaves were soaked in milliQ water and electrolyte leakage was measured after 2 h of incubation at room temperature with a conductivity meter (Horiba Ltd., Japan). The total electrolytes were determined after boiling for 10 min, and the extent of cell death was assessed with relative electrolyte leakage rates.

### Accession Numbers

The sequence data referred to in this manuscript can be found in the Arabidopsis Genome Initiative database under the following accession numbers: *ARPF2* (At3g23620), *ARRS1* (At2g37990), *RPL4A* (At3g09630), *APUM24* (At3g16810), and *PRH75* (At3g16810).

## Results

### Identification of the *Arabidopsis* Homolog of Yeast Rpf2

**FIGURE 1:**
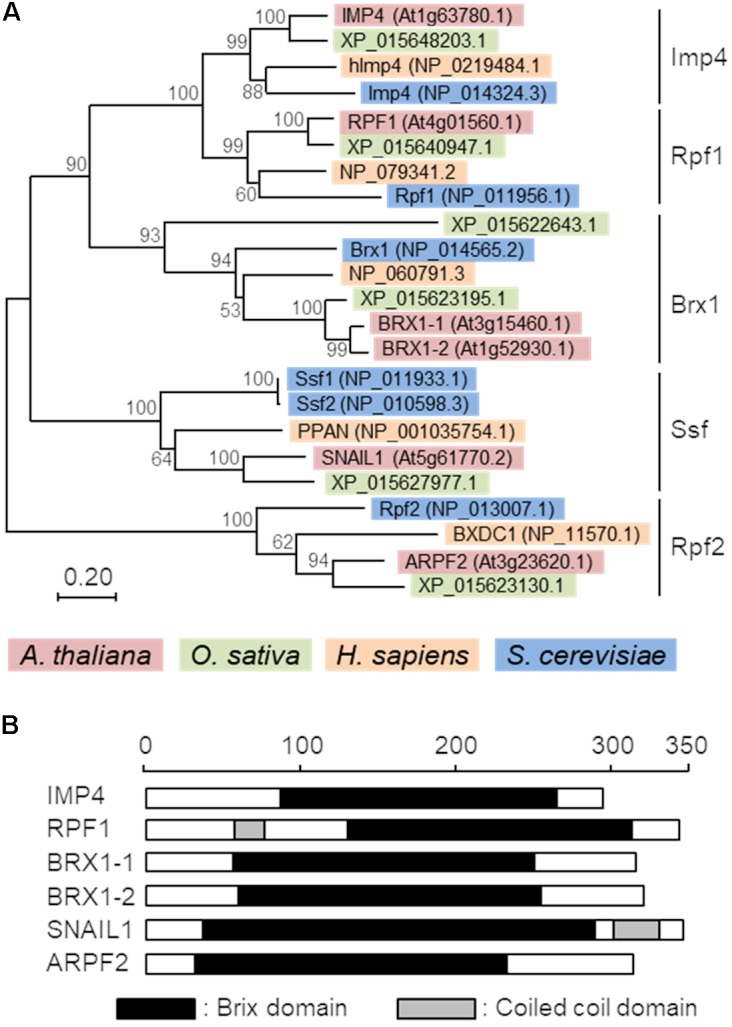
Phylogenetic tree and domain structures of Brix domain-containing proteins. **(A)** A phylogenic tree of Brix domain-containing proteins from *Arabidopsis thaliana*, *Oryza sativa, Homo sapiens*, and *Saccharomyces cerevisiae*. The percentage of replicate trees in which the associated taxa clustered together in the bootstrap test (1,000 replicates) is shown adjacent to the branches. The tree is drawn to scale, with branch lengths in the same units as those of the evolutionary distances used to infer the phylogenetic tree. **(B)** Schematic representation of the domain structures of six Arabidopsis Brix domain-containing proteins. Black and gray boxes indicate the Brix domains and coiled-coil domains, respectively. Protein architectures were deduced with SMART (Simple Modular Architecture Research Tool) (Letunic et al., 2015, http://smart.embl-heidelberg.de/). The scale indicates the number of amino acid residues.

The Brix domain-containing proteins are divided into five subgroups in yeast (Eisenhaber et al., 2001; Wehner and Baserga, 2002). A phylogenetic analysis of Brix domain-containing proteins from several organisms (*H. sapiens*, *A. thaliana*, *O. sativa*, and *S. cerevisiae*) revealed that all the analyzed organisms possess at least one member of each subgroup, suggesting that they play distinct but conserved roles in each organism (**Figure 1A**). Although the *Arabidopsis* proteins, BRX1-1 and BRX1-2, redundantly function (Weis et al., 2015b; Maekawa et al., 2018), similarity between two rice proteins in the Brx1 subgroup (XP_015622643.1 and XP_015623195.1) was low. Thus, it is currently uncertain as to whether these rice proteins play similar roles in rice. All the *Arabidopsis* Brix domain-containing proteins possess the Brix domain in the central region but have no other characteristic domain, except for the coiled-coil domain in ARPF1 and SNAIL1 (**Figure 1B**). Alignment of amino acid sequences of *Arabidopsis* Brix domains indicated that the overall homology is not high and that only several amino acid residues are conserved in them (**Supplementary Figure S1**). This observation may suggest that each Brix domain mediates intereactions with distinct interaction partners.

Of the six Brix domain-containing proteins in *Arabidopsis*, ARPF2 is the closest to the yeast Rpf2 in terms of amino acid sequences, with 35% identities and 52% similarities with 6% gaps in their amino acid sequence (**Supplementary Figure S2**). The co-expression analysis of *ARPF2* using the ATTEDII program (Obayashi et al., 2007) revealed that its expression pattern was similar to those of various ribosome-related genes including the other Brix domain-containing protein genes (**Supplementary Figure S3**), implying that *Arabidopsis* ARPF2 and yeast Rpf2 likely play a conserved role in ribosome biogenesis.

### *arpf2* Mutations Result in Aborted Embryos

**FIGURE 2:**
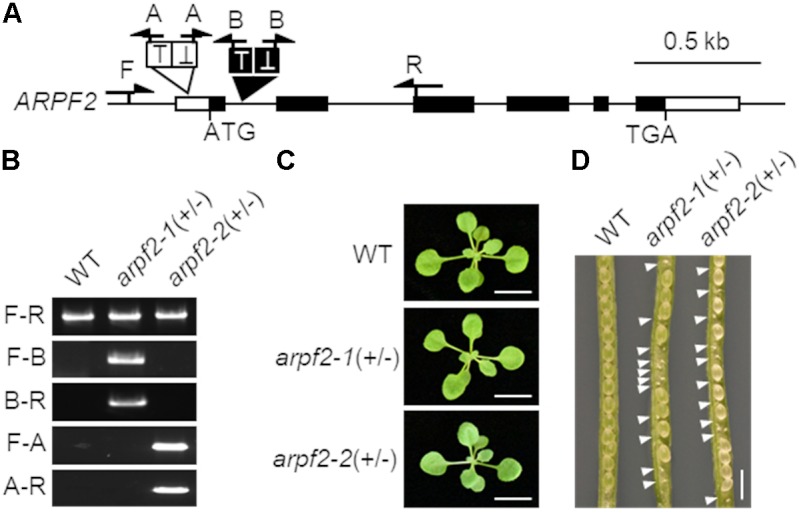
*arpf2* mutations lead to embryonic lethality in *Arabidopsis*. **(A)** Schematic representation of the *ARPF2* locus. White and black boxes indicate untranslated and coding regions in the exons of *ARPF2*, respectively. Black and white triangles indicate the positions of tandem T-DNA insertions in the *arpf2-1* and *arpf2-2* mutant alleles, respectively. Arrows indicate the positions of primers used for genotyping in **(B)**. **(B)** PCR-based genotyping. Wild type (WT) and heterozygotes for the *arpf2-1* or *arpf2-2* allele [*arpf2-1(+/–)* and *arpf2-2(+/–)*] were analyzed using primers (F, R, A, and B) indicated in **(A)**. Sets of primers F and R (F-R), primers F and A (F-A), primers A and R (A-R), primers F and B (F-B), and primers B and R (B-R) were applied. **(C)** Photographs of 2-week old plants grown on agar plates. Scale bars: 1 cm. **(D)** Photograph of seeds in fruits. White arrowheads indicate the aborted embryos. Scale bar: 1 mm.

To investigate the physiological role of ARPF2, we examined the phenotypes of two *Arabidopsis* T-DNA insertion lines in which the T-DNA insertions were located within the 1st intron (SALK_107828) and the 5’-UTR (SAIL_314_A03) of *ARPF2*. These mutant alleles were designated, *arpf2-1* and *arpf2-2* (**Figures 2A,B**). Although heterozygotes for *arpf2-1* and *arpf2-2* grew normally during the period for vegetative growth (**Figure 2C**), they developed aborted seeds (**Figure 2D**), suggesting that *arpf2-1* and *arpf2-2* are defective in gametogenesis and/or embryogenesis. Furthermore, we could not obtain homozygotes for the *arpf2-1* or *arpf2-2* allele. We, therefore, investigated the segregation ratio of the heterozygotes for *arpf2-1 and arpf2-2* in the progeny after self-pollination. In both the cases, the transmission efficiency of the *arpf2* mutation in the progeny population was much less than 100% (**Table 1**, χ^2^-test *p* < 0.001). Moreover, reciprocal crossing revealed that the transmission efficiency of the *arpf2* mutation was also much less than 100% (χ^2^-test *p* < 0.001) in a progeny population produced by crossing both the wild type with an *arpf2-1* heterozygote (**Table 1**). These results suggest that *ARPF2* is an essential gene involved in both female and male gametogenesis as well as in embryogenesis.

**Table 1:** Segregation of *rpf2-1* (+/-) and *rpf2-2* (+/-) self-progeny and reciprocal crosses between *rpf2-1* (+/-) and WT (+/+).

Parental genotypes (female x male)	Genotypes of F1 plants (n) of F1 plants (n)			Total (*n*)	Transmission efficiency (%)^a^	Chi-square test (*p*)
	+/+	+/-	-/-			
*rpf2-1 (+/-)* x *rpf2-1 (+/-)*	128	80	0	208	62.5	8.7E-04^b^ (vs 1:1)
*rpf2-2 (+/-)* x *rpf2-2 (+/-)*	50	22	0	72	44.0	9.7E-04^b^ (vs 1:1)
WT x *rpf2-1 (+/-)*	53	12	0	65	22.6	3.6E-07^b^ (vs 1:1)
*rpf2-1 (+/-)* x WT	60	7	0	67	11.7	9.4E-11^b^ (vs 1:1)


^a^Transmission efficiency (%) = (mutant/wild type) × 100. ^b^Significant difference (*p*< 0.05).

### *ARPF2* Is Expressed in a Variety of Tissues

**FIGURE 3:**
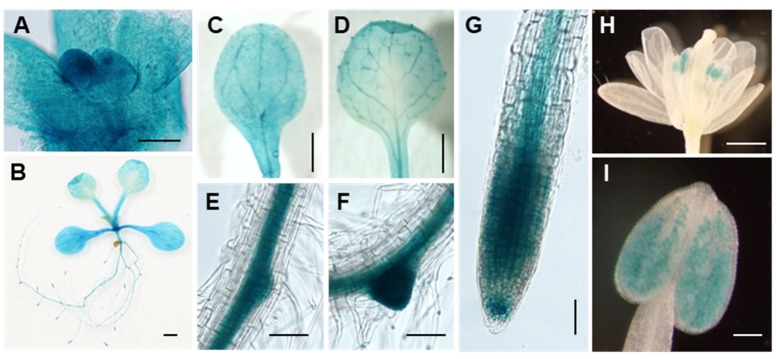
Tissue-specificity of *ARPF2* expression. Histochemical GUS staining of transgenic plants in which *GUS* expression was driven with the *ARPF2* promoter. Representatives of shoot apex of 2-day-old seedlings **(A)**, whole seedling **(B)**, cotyledon **(C)**, true leaf **(D)**, lateral root initiation **(E,F)**, and primary root tip **(G)** of 10-day-old seedlings, flower **(H)**, and pollen **(I)**. Three independent lines were analyzed with similar results. Scare bars: 50 μm in **(A)**, 1 mm in **(B–D,H)**, 100 μm in **(E–G,I)**.

To further characterize the ARPF2 gene, we examined the expression pattern of *ARPF2* using the GUS reporter gene. An approximately 1.5 kb region upstream of the *ARPF2* coding region was cloned and fused to the GUS reporter gene to make a construct, *pARPF2:GUS*, which was subsequently introduced into the wild type *Arabidopsis* (Col-0). Using T3 progenies of three independent transgenic lines harboring the *pARPF2:GUS* reporter construct, we checked the GUS expression in various tissues. In these three independent lines, the GUS expression patterns were similar. A typical expression pattern is shown in **Figure 3**. The *ARPF2* promoter-driven GUS reporter gene was highly expressed in the shoot apexes of 2-day-old seedlings (**Figure 3A**) and almost ubiquitously expressed in the seedlings grown for 10 days, especially in the vascular bundles in shoots and roots, and in the primary and lateral root primordia (**Figures 3B–G**). In the inflorescence, GUS activity was detected in the pollen (**Figures 3H,I**). These results suggest that the *ARPF2* promoter is probably active in a wide variety of tissues, particularly proliferating tissues, and therefore, the function of *ARPF2* appears to be fundamental to most of the cells.

### APRF2 Interacts With ARRS1 in the Nucleolus

**FIGURE 4:**
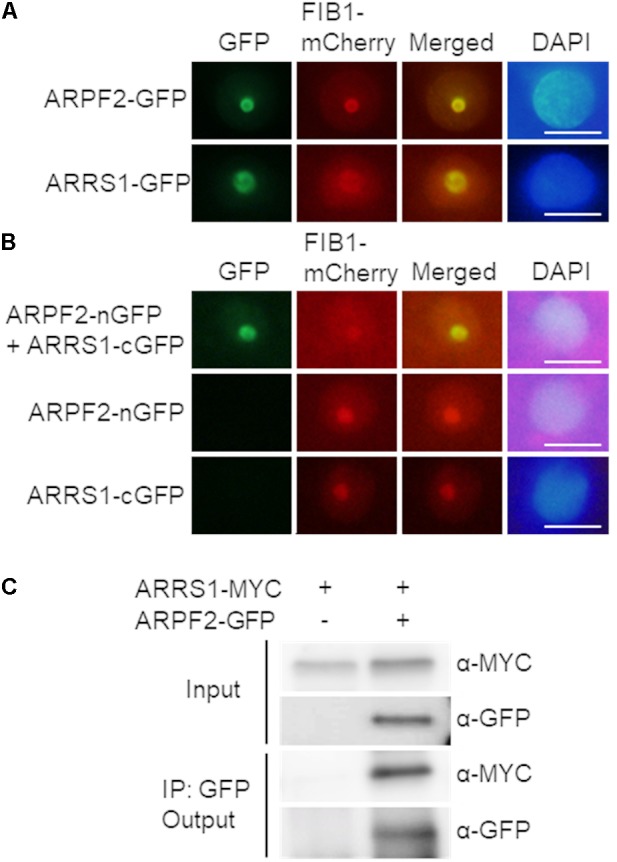
Interaction of ARPF2 with ARRS1 in the nucleolus. **(A)** Nucleolar localization of GFP fused to ARPF2 (ARPF2-GFP) and ARRS1 (ARRS1-GFP). Fluorescence from GFP and FIB1-mCherry (a nucleolar marker) was simultaneously monitored in *Nicotiana benthamiana* cells transiently expressing these proteins. Images for GFP were merged with those for fluorescence from FIB1-mCherry (Merged). DAPI staining indicates the nucleoplasm. Scale bars: 10 μm. **(B)** BiFC analyses for ARPF2 and ARRS1. ARPF2 fused to nGFP (ARPF2-nGFP) was co-expressed with cGFP-fused ARRS1 (ARRS1-cGFP) in *N. benthamiana* cells. The expression of cGFP and nGFP fused to the respective proteins alone served as negative controls. Scale bars: 10 μm. **(C)** Co-immunoprecipitation of ARPF2 and ARRS1. ARRS1-MYC was transiently expressed with or without ARPF2-GFP in *N. benthamiana* cells. The ARPF2-GFP protein in the crude cell extract (Input) was immunoprecipitated with an anti-GFP antibody, and the obtained precipitates (Output) were analyzed with anti-GFP and anti-MYC antibodies.

The cellular localization of ARPF2 was investigated by transiently expressing the ARPF2-GFP fusion protein in *N. benthamiana* leaves. Two days after the infection of *Agrobacterium* harboring the expression vector for ARPF2-GFP, the GFP fluorescence from ARPF2-GFP was detected only in the nucleolus. As shown in **Figure 4A**, the GFP fluorescence was perfectly merged with red fluorescence from mCherry fused to fibrillarin1 (FIB1), a nucleolus-localized marker protein (Barneche et al., 2000). The result indicates that like the yeast Rpf2 (Morita et al., 2002; Wehner and Baserga, 2002), ARPF2 is localized to the nucleolus.

In yeast, Rpf2 directly binds to Rrs1, a protein essential for the maturation of 25S rRNA and the 60S ribosomal subunit assembly (Morita et al., 2002), with high affinity. Although yeast Rpf2 can bind to 5S rRNA directly, Rpf2 more rigidly binds to 5S rRNA through forming the complex with Rrs1 (Madru et al., 2015). Furthermore, the Rpf2–Rrs1 complex is shown to be an essential component for docking 5S RNP into the pre-60S ribosome (Gambe et al., 2009; Madru et al., 2015). To investigate the Rpf2–Rrs1 interaction in plant cells, we identified the *Arabidopsis* protein homologous to the yeast Rrs1. In the BLAST search with the amino acid sequence for yeast Rrs1, only one *Arabidopsis* protein that had not been characterized yet (At2g37990.1) was found to display a high homology to yeast Rrs1 (34% identity, 54% similarity) (**Supplementary Figure S4**). Therefore, this protein is presumably the homolog of yeast Rrs1. This protein is referred to as *A. thaliana* homolog of yeast Rrs1 (ARRS1), hereafter. Rrs1 is localized to the nucleolus in yeast (Gambe et al., 2009). The nucleolar localization of ARRS1 was consistently confirmed by transient expression of the ARRS1-GFP fusion protein in *N. benthamiana* leaves (**Figure 4A**). To test the interaction between ARPF2 and ARRS1 *in vivo*, we firstly performed BiFC analysis with nGFP and cGFP (Walter et al., 2004). We observed the GFP fluorescence in the nucleolus after transient co-expression of the ARPF2-nGFP fusion protein with ARRS1-cGFP in *N. benthamiana* leaves (**Figure 4B**). No GFP fluorescence was detected in cells expressing ARPF2-nGFP or ARRS1-cGFP alone (**Figure 4B**). Similarly, no GFP fluorescence was detected in a control experiment using RPL4A, a component of the ribosome large subunit (Barakat et al., 2001), further supporting a specific interaction between ARPF2 and ARRS1 (**Supplementary Figure S5A**). Furthermore, we also detected the ARPF2–ARRS1 interaction by co-immunoprecipitation analysis with lysates of cells transiently expressing both the GFP-tagged ARPF2 (ARPF2-GFP) and MYC-tagged ARRS1 (ARRS1-MYC) (**Figure 4C**). The immunoblot analysis of the immunoprecipitates obtained using anti-GFP and anti-MYC antibodies demonstrated that these proteins were co-immunoprecipitated. Furthermore, specificity of the interaction between ARPF2 and ARRS1 was also supported by the result of a control co-immunoprecipitation using RPL4A as a negative control (**Supplementary Figure S5B**). These results strongly suggest a specific interaction between ARRS1 and ARPF2 in the nucleolus.

### ARPF2 Interacts With rRNAs *in planta*

**FIGURE 5:**
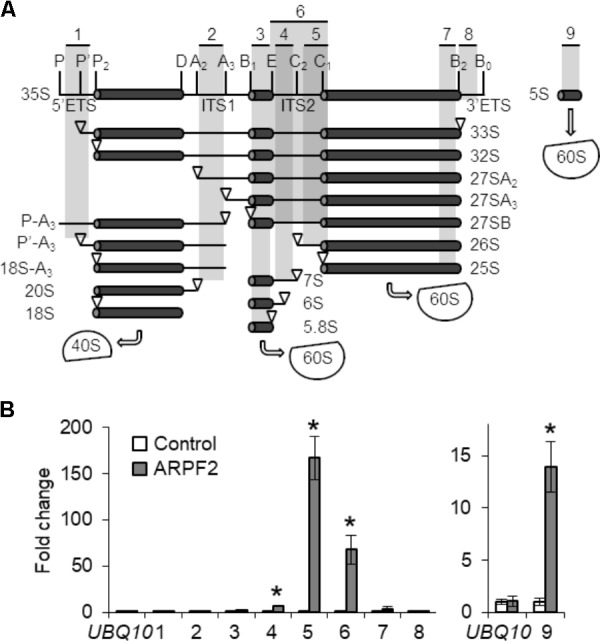
Binding of ARPF2 to pre-rRNA. **(A)** Scheme of the pre-rRNA processing pathway and processing intermediates in *Arabidopsis*. Vertical lines indicate the site of endo- or exonuclease processing steps. ETS and ITS are external and internal transcribed spacers, respectively. 40S and 60S are the 40S small subunit and the 60S large subunit, respectively. The positions of specific primers 1–9 used in **(B)** are indicated. **(B)** RT-qPCR analysis of RNA co-immunoprecipitated with wild type (Control) and ARPF2-GFP expressing transgenic (ARPF2) plants using anti-GFP antibodies. The enrichment of pre-rRNA or rRNA with specific regions [regions 1–9 in **(A)**] by co-immunoprecipitation was calculated with values obtained using the output sample versus those using the input sample. *UBQ10* is shown as a non-binding control. Error bars represent SD of three biological replicates. Asterisks indicate statistically significant differences between values obtained with the wild type (Control) and ARPF2-GFP expressors (ARPF2) by Student’s *t*-test (*p* < 0.05).

We investigated whether ARPF2 got bound to pre-rRNA, as yeast Rpf2 was shown to interact with 5S rRNA directly and with 25S rRNA directly or indirectly (Asano et al., 2015; Kharde et al., 2015; Madru et al., 2015). For this purpose, we performed RIP assay *in planta*. We prepared cell lysates of the wild type and ARPF2-GFP expressing plants and performed immunoprecipitation with anti-GFP antibodies after cross-linking the proteins to the RNA using formaldehyde and sonication to fragment the RNAs. Then, the levels of rRNA co-immunoprecipitated with ARPF2-GFP were quantified through RT-qPCR with several pairs of primers that allowed the amplification of different regions of pre-rRNAs or mature rRNA (**Figure 5A**). Among the various fragments originating from the rRNA, the fragments that contained ITS2 and 5S rRNA (primer sets 4, 5, 6, and 9) were significantly enriched, depending on the expression of ARPF2-GFP (**Figure 5B**). The result suggests *in vivo* interactions of ARPF2 with 5S rRNA and precursors of 25S rRNA that contained ITS2.

### *ARPF2* and *ARRS1* Overexpressors Show Shortened Stems, Abnormal Leaf Morphology, and Extension of Lifespan

**FIGURE 6:**
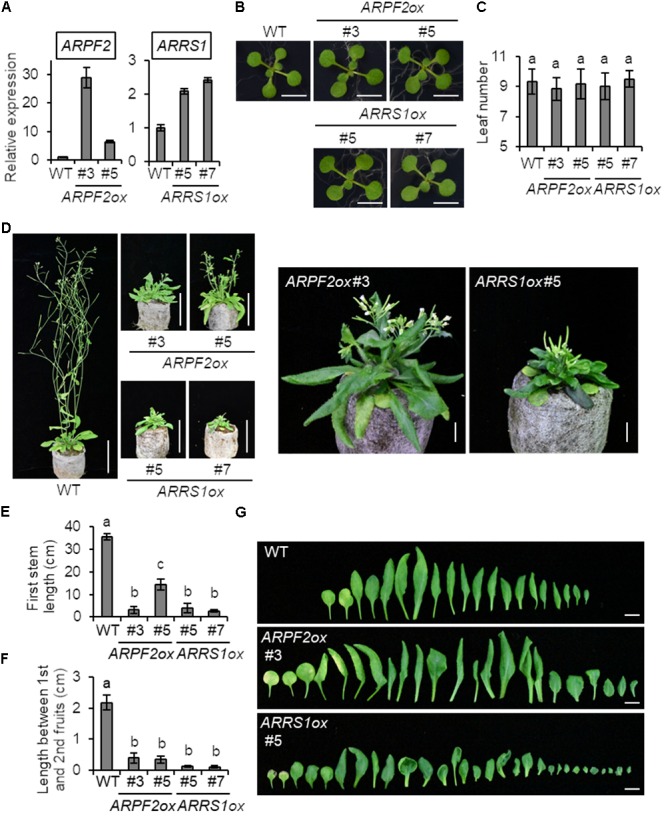
Phenotypic analysis of transgenic *Arabidopsis* overexpressing *ARPF2* or *ARRS1*. **(A)** Levels of *ARPF2* and *ARRS1* transcripts in the wild type and *ARPF2* or *ARRS1* overexpressing plants. RNA was extracted from 10-day-old seedlings and technical triplicates were analyzed for quantification. *UBQ10* was used as an internal control. The expression levels are relative to that in the wild type, and the values obtained with the wild type were set to 1. Error bars represent SD. **(B)** Photographs of 2-week-old seedlings grown on agar plates. Scale bars: 1 cm. **(C)** Numbers of rosette leaves at bolting. Error bars indicate SD (*n* = 6). **(D)** Photographs of the plants grown for 5-weeks. Scale bars: 5 cm. Enlarged view of *ARPF2ox* #3 and *ARRS1ox* #5 are also shown on the right side (scale bars: 1 cm). **(E,F)** Length of the first stem **(E)** and length between first and second fruits **(F)** of the plants grown for 5 weeks. Error bars indicate SD (*n* = 5). **(G)** Phenotype of rosette leaves of plants grown for 5 weeks. Scale bars: 1 cm. In **(C,E,F)** statistical significance was determined by ANOVA, followed by a Tukey–Kramer test under each experimental condition. Means that are significantly different from each other (*p* < 0.05) are indicated by different letters.

**FIGURE 7:**
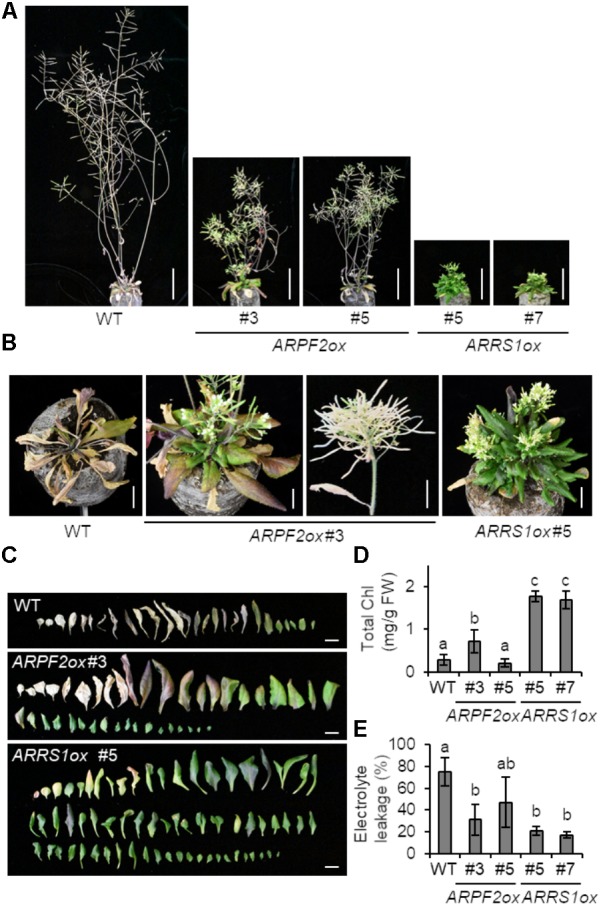
Extended lifespan of plants overexpressing *ARPF2* or *ARRS1*. **(A)** Photographs of the plants grown for 8 weeks. Scale bars: 5 cm. **(B)** Enlarged view of **(A)** (scale bars: 1 cm). **(C)** Phenotype of rosette leaves of plants grown for 5-weeks. Scale bars: 1 cm. **(D,E)** Quantification of total chlorophyll (Chl) **(D)** and cell death **(E)** of the plants grown for 8-weeks. The extent of cell death was assessed as relative electrolyte leakage (percentage of total electrolytes). Error bars indicate SD (*n* = 5). In **(D,E)**, the statistical significance was determined by ANOVA, followed by a Tukey–Kramer test. Means that are significantly different from each other (*p* < 0.05) are indicated by different letters.

To connect the function of ARPF2 with plant physiology, we generated transgenic *Arabidopsis* plants overexpressing *ARPF2* or *ARRS1*. Phenotypic analysis was performed with two independent transgenic lines in which the overexpression of *ARPF2* or *ARRS1* was confirmed (**Figure 6A**). We did not find any phenotypic characteristics of these overexpressors in the early period for vegetative growth (**Figure 6B**), and the number of rosette leaves at bolting indicated that these overexpressors were comparable to the wild type in terms of the timing of flowering (**Figure 6C**). However, the 5-week-grown *ARPF2* and *ARRS1* overexpressors similarly displayed shorter stem than those of the wild type (**Figures 6D,E**), accompanied with the shortened lengths of internodes (**Figures 6D,F**). At this stage, the abnormality in the leaf shape became evident (**Figures 6D,G**). The height of the well-maturated overexpressors grown for 8-weeks were still shorter than those of the wild type plants (**Figure 7A**). In addition to this phenotypic characteristic, 8-week-old overexpressors were still in the growth phase, accompanied with living leaf cells containing a certain amount of chlorophyll, whereas the wild type plants were almost completely withered (**Figures 7A–E**). However, these overexpressors finally developed viable seeds. Because the overexpression of *ARPF2* and *ARRS1* had no effect on the timing of flowering (**Figure 6C**), it was unlikely that *ARPF2* and *ARRS1* overexpression caused a delay in growth and then extended the total length of life. Hence, these observations suggest that the overexpression of *ARPF2* or *ARRS1* greatly affected the growth in the reproduction phase and extended their lifespan.

To examine whether the overexpression of other ribosome biogenesis-related genes similarly induced the same phenotype, we produced transgenic *Arabidopsis* overexpressing *APUM24* or *PRH75* (**Supplementary Figure S6**). *APUM24* encodes a pre-rRNA processing factor that plays an essential role in removing ITS2 through the interaction with ITS2 (Shanmugam et al., 2017; Maekawa et al., 2018), whereas *PRH75*/At*RH7* encodes a DEAD-box RNA helicase involved in rRNA processing (Nayak et al., 2013; Weis et al., 2015b; Huang et al., 2016). The growth of these overexpressors was comparable to that of the wild type (**Supplementary Figure S6**), indicating that the overexpression of ribosome biogenesis-related genes does not always induce phenotypes found in the *ARPF2* and *ARRS1* overexpressors, such as short stem, abnormal leaf morphology, and extended lifespan.

## Discussion

Based on the findings in the current study, we conclude that ARPF2 is the *Arabidopsis* homolog of yeast Rpf2 and plays an essential role in ribosome biogenesis through its interactions with the *Arabidopsis* homolog of yeast Rrs1 (ARRS1), 5S rRNA, and 25S RNA precursors containing ITS2. We also suggest that the overexpression of *ARPF2* or *ARRS1* leads to unique phenotypic characteristics that are not produced by overexpressing other ribosome biogenesis-related genes.

The Brix domain-containing proteins can be divided into five subgroups that are probably responsible for distinct biological processes (**Figure 1**). Consistent with this idea, the double mutant of two *Arabidopsis* genes belonging to the Brx1 subgroup could not survive (Weis et al., 2015b). Furthermore, function-deficient mutants of a unique gene of the Ssf subgroup also showed lethality (Hao et al., 2017). Similarly, our finding of embryonic lethality in the *arpf2* mutant (**Figure 2** and **Table 1**), which was most likely caused by the ribosome biogenesis defects, also supports the idea that proteins in the distinct subfamily of the Brix domain protein family have essential but distinct roles.

By revealing the interactions of ARPF2 with ARRS1 in the nucleolus (**Figure 4**) and specific regions of rRNAs and/or their precursors *in vivo* (**Figure 5**), we also suggest that the function of ARPF2 is similar to those of yeast Rpf2 and its human homolog, BXDC1, in ribosome biogenesis. The function of the Rpf2–Rrs1 complex was proposed as a requirement of the 5S RNP (5S rRNA–Rpl5–Rpl11 complex) into the central protuberance of the pre-60S ribosome particles to correctly assemble the 5S RNP with the pre-60S ribosome particles in the nucleolus (Madru et al., 2015). In fact, depletion of Rpf2 led to a reduction in the rate of conversion of 27SB pre-rRNA to the mature 25S rRNA and in the impairment of nuclear export of pre-ribosomes (Wehner and Baserga, 2002; Zhang et al., 2007). Similarly, the complex of BXDC1 and hRRS1, human homolog of Rrs1, were proposed to mediate the assembly of the 5S RNP into the pre-ribosome particle, and depletion of BXDC1 caused processing defect of pre-rRNA in the large ribosomal subunit, mislocalization of Rpl5 and Rpl11 ribosomal proteins, and loss of nucleolar structure integrity (Donati et al., 2013; Sloan et al., 2013; Nicolas et al., 2016). Although we suggest that ARPF2 and ARRS1 constitute the 5S RNP in *Arabidopsis*, our results do not perfectly match with those of the published reports. The yeast Rpf2 has been shown to interact not only with 5S rRNA but also with 7S rRNA (Asano et al., 2015; Kharde et al., 2015; Madru et al., 2015). In another report, Rpf2 was also shown to bind to 35S, 27SA, and 27SB rRNAs *in vivo* (Wehner and Baserga, 2002; Zhang et al., 2007). The results of our RIP assay indicated the binding of ARPF2 to 5S rRNA and ITS2 that is present in the 25S RNA precursors (35S, 33S, 32S, 27SA, 27SAB, and 26S RNAs) but not in 25S RNA (**Figure 5**). Although our results do not rule out the binding of ARPF2 with 25S rRNA, the suggested regions recognized by ARPF2 and Rpf2 are subtly different. The exact identification of recognition sites of the Rpf2 subfamily proteins would be necessary to fully understand the Rpf2–Rrs1 complex-mediated process in ribosome biogenesis.

Very intriguingly, the overexpression of *ARPF2* and *ARRS1* did not induce any apparent phenotype in the early period for vegetative growth but caused drastic morphological changes, including short stem and abnormal leaf morphology, in the late period of vegetative growth and in the period for reproductive growth (**Figures 6, 7**). Furthermore, as the most interesting phenotypic characteristic, ARPF2- and ARRS1-overexpressors showed the extension of lifespan because of the extended period for reproductive growth (**Figure 7**). Overexpressing other ribosome-related factor genes (*APUM24* and *PRH75*) did not give rise to such phenotypes (**Supplementary Figure S6**). Hence, these phenotypes appear to be due to the effects that are specifically linked to the overexpression of *ARPF2* and *ARRS1*, although this possibility should be further examined in future. The molecular mechanism underlying these phenotypes is uncertain at this stage; however, we speculate that aberrant expression levels of *ARPF2* and *ARRS1* might generate incompleted forms of the ARPF2- and ARRS1-containing complexes that induce production of the unprocessed or miss-processed rRNA-containing ribonucleoprotein complexes and unexpected phenotypes. This highly speculative hypothesis should also be verified in future.

Although it is currently unknown whether the overexpression of the counterparts of *ARPF2* or *ARRS1* also induces a similar phenotype in animals or yeast, overexpressing the other rRNA processing factors, Dkc1 and Rrp5, has also been shown to extend the lifespan of mouse embryonic fibroblasts through an unknown mechanism (Nishimura et al., 2015). In the cells overexpressing Dkc1 or Rrp5, various cellular senescence-associated phenomena, including increases in the amount of nucleolar rRNA and the enlargement of nucleolus, occurred, accompanied with reduction in the amounts of p53, p21, and p16 that are key factors for cell senescence (Nishimura et al., 2015). Taken together with our findings, it is likely that the lifespan extension is selectively caused by the overexpression of particular rRNA processing factors in both animals and plants. Further analysis of the molecular mechanisms underlying this phenomenon would assist in fully understanding the ribosome biogenesis and might also provide a clue about the mechanism determining the lifespan of cells and living organisms.

Many ribosome-related genes have been characterized with loss-of-function mutants, thus far (reviewed in Horiguchi et al., 2012 and Weis et al., 2015a). However, it is worth pointing out that the findings in the current study highlight the potential usefulness of the plants overexpressing the ribosome-related genes for new valuable discoveries.

## Author Contributions

SM and SY designed the research, analyzed the data and wrote the article. SM and YU performed the experiments.

## Conflict of Interest Statement

The authors declare that the research was conducted in the absence of any commercial or financial relationships that could be construed as a potential conflict of interest.
