# Supplementary material for: Overexpression of a Brix Domain-Containing Ribosome Biogenesis Factor ARPF2 and its Interactor ARRS1 Causes Morphological Changes and Lifespan Extension in Arabidopsis thaliana
Source: Front Plant Sci. 2018 Aug 27;9:1177. doi: 10.3389/fpls.2018.01177 (PMC6120060; doi:10.3389/fpls.2018.01177)
Supplement: Supplementary file 1 [file Presentation_1.pdf]

```
IMP4      LLTTSRNPSAPLIRFTKELKFVFP------NSQR---INRGSQVISEIIETARSHDFTDVILVHEHRGVPDGLIISHLPFGPTAYFGLLNVVTRHDISDK 91
RPF1      LLTTCRFNSTRGPALISELLSVIP------NSHY---QKRGTYDLKKIVEYATKKDFTSLIVVHTNRREPDALLIIGLPNGPTAHFKLSNLVLRKDIKN- 90
BRX1-1    LVTCSRRINFRYRHLMLNMVSLLP------HCKKDSKVEAKSSRGATLNELIELKGSSSCLFFECRKHKDLYMWMVKSPGGPSVKFLVNAVHTMEELKLT 94
BRX1-2    LVTCSRRISFRYRSLMLNIVSLLP------HCKKDSKVEAKSSKGATLNELIELKNSNSCLFFECRKHKDLYMWMVKSPNGPSVKFLVKAVHAMEEMKLT 94
SNAIL1    FVFSRMKLAGPVKQLQMDLRKLML------PYTALSLKEKKRNTLRDFLNVSGPMGVTHFLMLSKTAS-SLSLRVARTPQGPTLTFKIHQYSLASDIAQS 93
ARPF2     LILHGTKTSATLSSVMTELYRLKKGGAIRYSRRNENIRPFESGGETSLEFFSQKTDCSIFVYGSHTKKRPDNLVLGRMYDHQVYDLIEVGIENFKSLRAF 100

IMP4      KSIGKMPE------QYPHLIFN-NFTTQMGQRVGNILKHIFP-APKLDAKR-----IVTFSNQSD--YISFRNHVY------------------------ 152
RPF1      --HGNPTS------HQPELVLN-NFTTRLGNRVGRFFQSLFPPDPNFRGRR-----VVTFHNQRD--FIFFRHHRYIFETKESK---------------- 158
BRX1-1    GNHLKGSR------PLLTFSSN-FENDAHWKLLKEMLTQIFGIPEGHRKSKPYHDHVFVFSIVDD--HIWFRNYQISVPHNES----------------- 168
BRX1-2    GNHLKGSR------PLLTFSSN-FDKDAHWKLLKEMLTQVFGIPKEHRKSKPYHDHVFVFSIVDE--HIWFRNYQISVPHNES----------------- 168
SNAIL1    QLRPRCPQDLFKSPPLIVLSGF-GSQELHLKLATIMFQNIFPAIDINTVKLSTCQRLVLLNYNKDTKLIDFRHYSIRLQPVGVSRRIRKFVQNHQVPDLR 192
ARPF2     SYDKKFAPHEG-TKPFICFIGEGFENVSELKHLKEVLTDLFRGEVVDNLNLTGLDRAYVCSAISP-TKVFLTHCALKLK--------------------- 177

IMP4      ----------------------------------DKGEGGPK-SIELKEIGPRFELRLYQV 178
RPF1      ---------------------------------SDKGKEETI-KPRLQECGPRFTLKLVTL 185
BRX1-1    ---------------------------------DKIARGDLD-KMTLIEVGPRFCLNPIKI 195
BRX1-2    ---------------------------------DKIAKGGLD-KMTLIEVGPRFCLNPIKI 195
SNAIL1    NLQDVSDFVTKAGYGSESEGDEEAATVTLSSDLGRVNKGATKSAVKLQEIGPRMTMQLVKV 253
ARPF2     ------------------------------------KSGSIVPRMELVEVGPSMDLVIRRN 202
```

Figure S1 Alignment of amino acid sequences of Brix domains from *Arabidopsis thaliana*. The sequence alignment was produced with the ClustalW program (http://www.genome.jp/tools/clustalw/). Amino acid residues conserved in all amino acid sequences are shaded in gray.

Supplemental Figure S1
Maekawa et al.

```
ARPF2  MMEIRTPKTGKAKRVLESRAPKLVETGKKTLILHGTKTSATLSSVMTELYRLKKGGAIRY 60
 Rpf2  MIRTVKPKNARAKRALVKREAKLVENVKQALFIPGQSCNKNLHDIMVDLSALKKPDMKRF 60

ARPF2  SRRNENIRPFESGGETSLEFFSQKTDCSIFVYGSHTKKRPDNLVLGRMYDHQVYDLIEVG 120
 Rpf2  NRKND-IHPFED--MSPLEFFSEKNDCSLMVLMTSSKKRKNNMTFIRTFGYKIYDMIELM 117

ARPF2  IENFKSLRAFSYDKKFAPHEGTKPFICFIGEGFENVSELKHLKEVLTDLFRGEVVDNLNL 180
 Rpf2  VADN--FKLLSDFKKLTFTVGLKPMFTFQGAAFDTHPVYKQIKSLFLDFFRGESTDLQDV 175

ARPF2  TGLDRAYVCSAISPT-------KVFLTHCALKLKKS---GSIVPRMELVEVGPSMDLVIR 230
 Rpf2  AGLQHVISMTIQGDFQDGEPLPNVLFRVYKLKSYKSDQGGKRLPRIELVEIGPRLDFKIG 235

ARPF2  RNRLPNDSLMKEAMRTSKDKPKKKEKNVDQDAVLGKTGKIYMPDQ-----KLKEMKLFDK 285
 Rpf2  RIHTPSPDMVTEAHKKPKQLEMKTKKNVELDIMGDKLGRIHMGKQDLGKLQTRKMKGLKS 295

ARPF2  SKGSKRERKDAKLKHKEETVAKKMKVSSE-------------------- 314
 Rpf2  KFDQGTEEGDGEVDEDYEDEASYSDDGQEYEEEFVSATDIEPSAKRQKK 344
```

Figure S2 Alignment of amino acid sequences of ARPF2 and Rpf2. The sequence alignment of ARPF2 (At3g23620.1) and *Saccharomyces cerevisiae* Rpf2 (NP_013007.1) was produced with the ClustalW program (http://www.genome.jp/tools/clustalw/). Similar and identical amino acid residues in the two amino acid sequences are shaded in light and dark gray, respectively. The sequences of the Brix domain are indicated by red line.

Supplemental Figure S2
Maekawa et al.

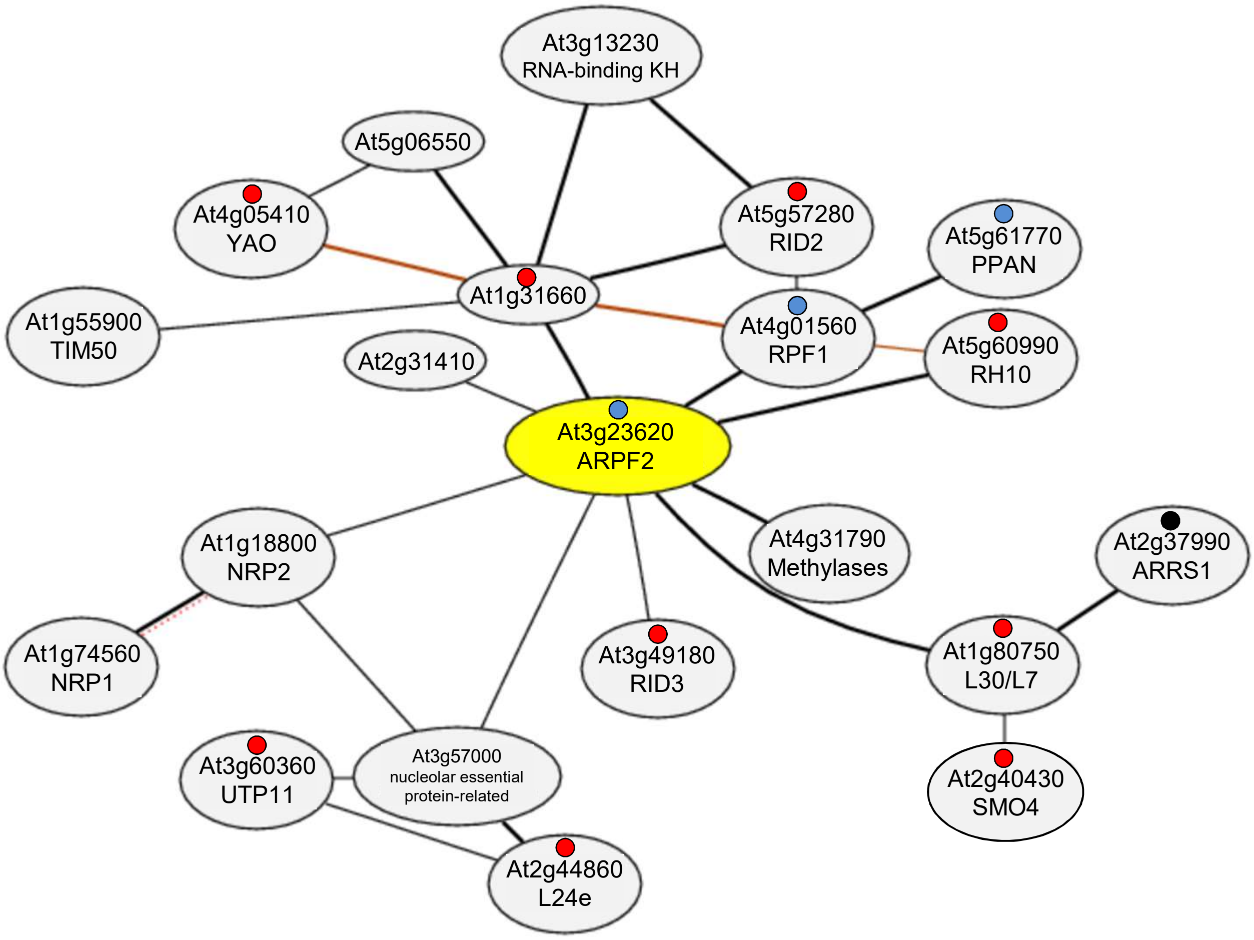

Figure S3 Co-expression network including *ARPF2*. A co-expression network of *ARPF2* was obtained using the ATTEDII program (http://atted.jp/) with the parameter "Coexpression data; All". Genes reported as "ribosome-related gene" are indicated by red dots; At1g31660, Missbach et al. (2013); *RID2* (At5g57280) and *RID3* (At3g49180), Shinohara et al. (2014); *YAO* (At4g05410), Li et al. (2010); *L24e* (At2g44860) and *L30/L7* (At1g80750), Carroll et al. (2008); *RH10* (At5g60990), Matsumura et al. (2016); *SMO4* (At2g40430), Zhang et al. (2015). Genes encoding Brix domain-containing proteins are indicated by blue dots. *ARRS1* is indicated by black dot.

Supplemental Figure S3
Maekawa et al.

```
ARRS1  --------MDTEMETEQIYQVDVGNLLAFNP----NHRFPSAPSSRGELVKEILTEGTKL 48
 Rrs1  MSAEDYKNLPVTVEKPIPVVYDLGNLAAFDSNVLDKNDLDSSNARREEKIKSLTRDNVQL 60

ARRS1  VQEIANKLFNFPSTETNDGP---------IVQLPPPTTKLPREKHIPRPKPPTKWEEFALK 100
 Rrs1  LINQLLSLPMKTTTESVGGTGGQSSVMTLLQLPDPTTDLPREKPLPKAKAMTKWEKFAAK 120

ARRS1  KGIQKRKKE-KVVWDEQTNQFKRRHGYDRVNDDNDVPIIEAKESDEPG-----VDPFAKR 154
 Rrs1  KGIKPKERAGKMIYDEASGEWVPKWGYKGANKKLDDQWLVEVDDKVKGTDNELIDPRTLN 180

ARRS1  LDDKKKRVGKQEKNRLQNLKAAEKAGALPSHVQLAATSLPISGTKAQPKKIGKDELGDVA 214
 Rrs1  RAERKRLVKKNEKQQRRNMKNAL------------------------------------- 203

ARRS1  GLAATSTASGGKFDKKLPGEKPPKKQGKHHKYLPVVSGRGDVNAEKEQTNNVLSKIFSKH 274
 Rrs1  ------------------------------------------------------------

ARRS1  SHEILNVGKAINMYNVKKEKKKSGRSDKLKPKKDITKKPANKAK 318
 Rrs1  --------------------------------------------
```

Figure S4 Alignment of amino acid sequences of ARRS1 and Rrs1. Sequence alignment of ARRS1 (At2g37990.1) and *Saccharomyces cerevisiae* Rrs1 (NP_014937.1) was produced with the ClustalW program (http://www.genome.jp/tools/clustalw/). Similar and identical amino acid residues in the two amino acid sequences are shaded in light and dark gray, respectively.

Supplemental Figure S4
Maekawa et al.

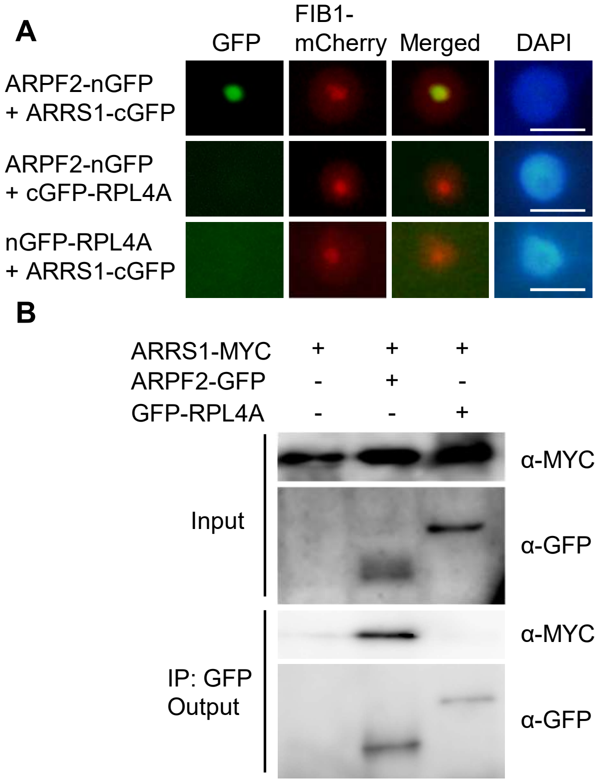

Figure S5 Specificity of the ARPF2- ARRS1 interaction. (A) BiFC assays. ARPF2 fused to nGFP (ARPF2-nGFP) was co-expressed with cGFP-fused ARRS1 (ARRS1-cGFP) or RPL4A (cGFP-RPL4A) and FIB1-mCherry in *N. benthamiana* leaves. ARRS1-cGFP was also co-expressed with GFP-fused RPL4A (nGFP-RPL4A) and FIB1-mCherry. Scale bars: 10 µm. (B) Co-immunoprecipitation assays. ARRS1-MYC was transiently expressed alone or with ARPF2-GFP or GFP-RPL4A in *N. benthamiana* leaves. The ARPF2-GFP or GFP-RPL4A proteins in the crude cell extracts (Input) were immunoprecipitated with an anti-GFP antibody, and the obtained precipitates (Output) were analyzed with anti-GFP and anti-MYC antibodies.

Supplemental Figure S5
Maekawa et al.

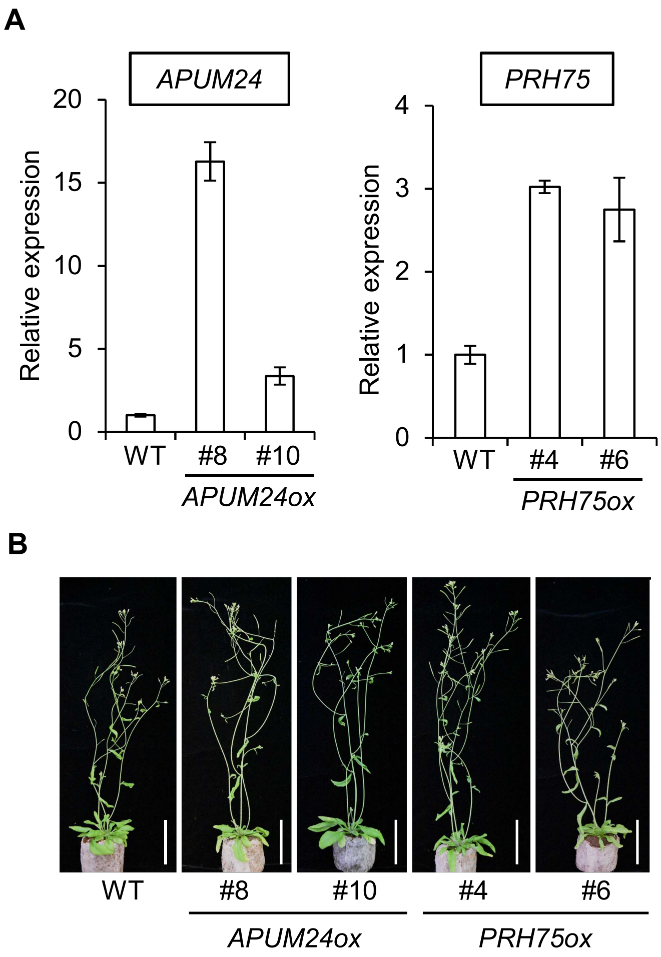

Figure S6 Phenotypic analysis of transgenic *Arabidopsis* overexpressing *APUM24* or *PRH75*. (**A**) Levels of *APUM24* and *PRH75* transcripts in the wild type and *APUM24* or *PRH75* overexpressing plants. RNA was extracted from 10-day-old seedlings and technical triplicates were used for quantification. *UBQ10* was used as an internal control. The expression levels are relative to that in the wild type, and the values obtained with the wild type were set to 1. Error bars represent SD. (**B**) Photographs of the plants grown for 5 weeks. Scale bars: 5 cm.

Supplemental Figure S6
Maekawa et al.

Supplemental Table S1. List of the Primer Sequences.

Primer name	Sequence (5'-3')
Vector construction	
pRPF2 5'entry	CACCACCTATAGAGACGACAATGTC
pRPF2 3'entry	GACTCTGTTAAATCGCCGGAAAG
RPF2 5'entry	CACCATGATGGAAATACGAACTCC
RPF2 3'entry	CTCAGAAGAGACCTTCATCTTTTTGG
RRS1 5'entry	CACCATGGACACGGAGATGG
RRS1 3'entry	CTTGGCTTTGTTGGCAGGCTTC
APUM24 5'entry	CACCATGTCTTCCAAAGG
APUM24 3'entry	TTCAGGTTTCTTGGTTGCTGAGATC
PRH75 5'entry	CACCATGCCTTCCCTAATGTTATCTG
PRH75 3'entry	TCAATATCTCTGGCCTCTACCACCAC
RPL4A 5'entry	CACCATGGCCGCCGCCGCT
RPL4A 3'entry	TTACTGGCTAGCACCGAGCCATTTG
Genotype check	
Primer "A"	TGGTTCACGTAGTGGGCCATCG
Primer "B"	TTCATAACCAATCTCGATACAC
Primer "F"	GTCACTATAGTTGTTGCTTACCTCATG
Primer "R"	GTCGTTTCTTCGTGTGAGAACC
RT-qPCR	
RPF2 F	CATCCAAGGATAAGCCTAAGAAGAAG
RPF2 R	CTCAGAAGACACCTTCATCTTTTTG
RRS1 F	TGGGGATGTTAACGCAGAAAAAGAAC
RRS1 R	CTTGGCTTTGTTGGCAGGCTTC
APUM24 F	CTTGTCAACAGTGGCCTTG
APUM24 R	TCACACAGCTTCTCGCTCAG
PRH75 F	GAGGATTCTACTAAAGTCCAGAC
PRH75 R	ATAATATCAGGAATCAACCGAGCC
UBQ10 F	GATCTTTGCCGGAAAACAATTGGAGGATGGT
UBQ10 R	CGACTTGTCATTAGAAAGAAAGAGATAACAGG
Forward primer of "1" in Fig. 5	TTCGGCGTATGAGTGGTG
Reverse primer of "1" in Fig. 5	CATTCATCGATCACGGCAATTCC
Forward primer of "2" in Fig. 5	AACGACCCGCGAACCAAAGATCAC
Reverse primer of "2" in Fig. 5	ACACTTTTCGTGCCGGGGTTTTGTG
Forward primer of "3" in Fig. 5	CGACTCTCGGCAACGGATAT
Reverse primer of "3" in Fig. 5	TTGTGACACCCAGGCAGACG
Forward primer of "4" in Fig. 5	ATCGTCGTCCCTCACCATCCTTTGCTGATG
Reverse primer of "4" in Fig. 5	GCATGTCGGTACGCTCCA
Forward primer of "5" in Fig. 5	TGATCCATTACATTTTATCGGTCGC
Reverse primer of "5" in Fig. 5	CTTAAACTCAGCGGGTAATCC
Forward primer of "6" in Fig. 5	CACGTCTGCCTGGGTGTCAC
Reverse primer of "6" in Fig. 5	Same as reverse primer of "5" in Fig. 5
Forward primer of "7" in Fig. 5	AATCCGGGCTAGAAGCGACG
Reverse primer of "7" in Fig. 5	AATTCAAGGCGGTCCGAACGAC
Forward primer of "8" in Fig. 5	GCCCTTTGTCGCTAAGATTCGA
Reverse primer of "8" in Fig. 5	GTTTTGGACAAGTCTGTCTCTTCAC
Forward primer of "9" in Fig. 5	GATGCGATCATACCAGCACTAATGC
Reverse primer of "9" in Fig. 5	AGGGATGCAACACGAGGACTTC
